# Incremental substitution of Ni with Mn in NiFe_2_O_4_ to largely enhance its supercapacitance properties

**DOI:** 10.1038/s41598-020-67802-z

**Published:** 2020-07-02

**Authors:** Samira Sharifi, Ahmad Yazdani, Kourosh Rahimi

**Affiliations:** Condensed Matter Group, Department of Basic Sciences, Tarbiat Modares University, Jalal-Ale-Ahmad Avenue, Tehran, Iran

**Keywords:** Energy storage, Condensed-matter physics

## Abstract

By using a facile hydrothermal method, we synthesized Ni_1−x_Mn_x_Fe_2_O_4_ nanoparticles as supercapacitor electrode materials and studied how the incremental substitution of Ni with Mn would affect their structural, electronic, and electrochemical properties. X-ray diffractometry confirmed the single-phase spinel structure of the nanoparticles. Raman spectroscopy showed the conversion of the inverse structure of NiFe_2_O_4_ to the almost normal structure of MnFe_2_O_4_. Field-emission scanning electron microscopy showed the spherical shape of the obtained nanoparticles with a size in the range of 20–30 nm. Optical bandgaps were found to decrease as the content of Mn increased. Electrochemical characterizations of the samples indicated the excellent performance and the desirable cycling stability of the prepared nanoparticles for supercapacitors. In particular, the specific capacitance of the prepared Ni_1−x_Mn_x_Fe_2_O_4_ nanoparticles was found to increase as the content of Mn increased, reaching the highest specific capacitance of 1,221 F/g for MnFe_2_O_4_ nanoparticles at the current density of 0.5 A/g with the corresponding power density of 473.96 W/kg and the energy density of 88.16 Wh/kg. We also demonstrated the real-world application of the prepared MnFe_2_O_4_ nanoparticles. We performed also a DFT study to verify the changes in the geometrical and electronic properties that could affect the electrochemical performance.

## Introduction

Supercapacitors have attracted a lot of attention as an energy storage alternative for batteries due to their potentially promising applications in hybrid electric vehicles, mobile electronic devices, and memory backup systems thanks to their ability in providing high power and energy densities simultaneously^1–3^. Supercapacitors, also known as electrochemical capacitors, are generally classified into two categories according to their charge storage mechanisms^4,5^. The first category is the so-called electric double-layer capacitor (EDLC), where the capacitance arises from the electrostatic charge storage at the interfaces between the electrodes and the electrolyte^6^. The second category is the so-called pseudocapacitor, which involves faradaic reactions but behaves like a capacitor, not a galvanic cell. The typical capacitance per unit surface area of the electrode material in pseudocapacitors is more than 10 times that in EDLCs^7^.

Transition metal oxides are promising materials in photocatalysts, sensors, solar cells, etc.^8–13^, and especially as supercapacitor electrodes thanks to their fast, reversible faradaic redox reactions (or pseudocapacitance)^14–20^. For example, mixed metal oxides including Ni/Mn, Mn/Fe, and Mn/Ni/Co, Ni/Co/Mo oxides have significantly improved the electrochemical performance^21–24^. Nowadays, designing complex metal oxide-based materials with high specific capacitance and a low cost is desirable. Among the metal oxides, spinel ferrites are fascinating due to their impressive magnetic, electrical, and optical properties as well as their ability to exhibit different redox states and electrochemical stability^25,26^. It is expected that the ferrites could offer richer redox reactions, including contributions from both M and Fe ions (in MFe_2_O_4_) than those of the single-metal oxides^27^. Binary metal oxide ferrites (MFe_2_O_4_) have been widely studied for supercapacitor applications including MFe_2_O_4_ (M = Fe, Co, Ni, Mn, Cu, Zn)^28^, NiCo_2_O_4_^29^, MnFe_2_O_4_^7,30^, and CoFe_2_O_4_^27^. Spinel ferrites may also consist of a mixture of two divalent metal ions, in which the ratio of these divalent ions may vary, and they are referred to as mixed ferrites. The cation distribution of the mixed ferrites significantly affects their surface properties, making them catalytically active. Therefore, it is worthwhile investigating the suitability of mixed ternary-transition-metal ferrites (A_x_B_1−x_Fe_2_O_4_) in the challenging field of supercapacitors. For example, it has been shown that CuCoFe_2_O_4_, NiCoFe_2_O_4_, and NiCuFe_2_O_4_ can be promising for supercapacitors because of their low cost and low toxicity^25^. Furthermore, there are some reports on MnCoFeO_4_^31^, MnZnFe_2_O_4_^32^, and thin films of Ni_x_Mn_1−x_Fe_2_O_4_ (x = 0.2, 0.4, 0.6, 0.8)^33^ as supercapacitor electrodes. However, mixed ternary-transition-metal ferrites deserve more investigation to enter the commercial real-world applications.

Here, we carried out a systematic study to see how the incremental substitution of Ni with Mn in hydrothermally synthesized Ni_1−x_Mn_x_Fe_2_O_4_ nanoparticles will affect their structural, electronic, and electrochemical properties. Finally, we performed a density functional theory study on the same structures to confirm the changes in their geometrical, electronic, and electrochemical properties.

## Experimental methods

### Synthesis procedure

**Figure 1:**
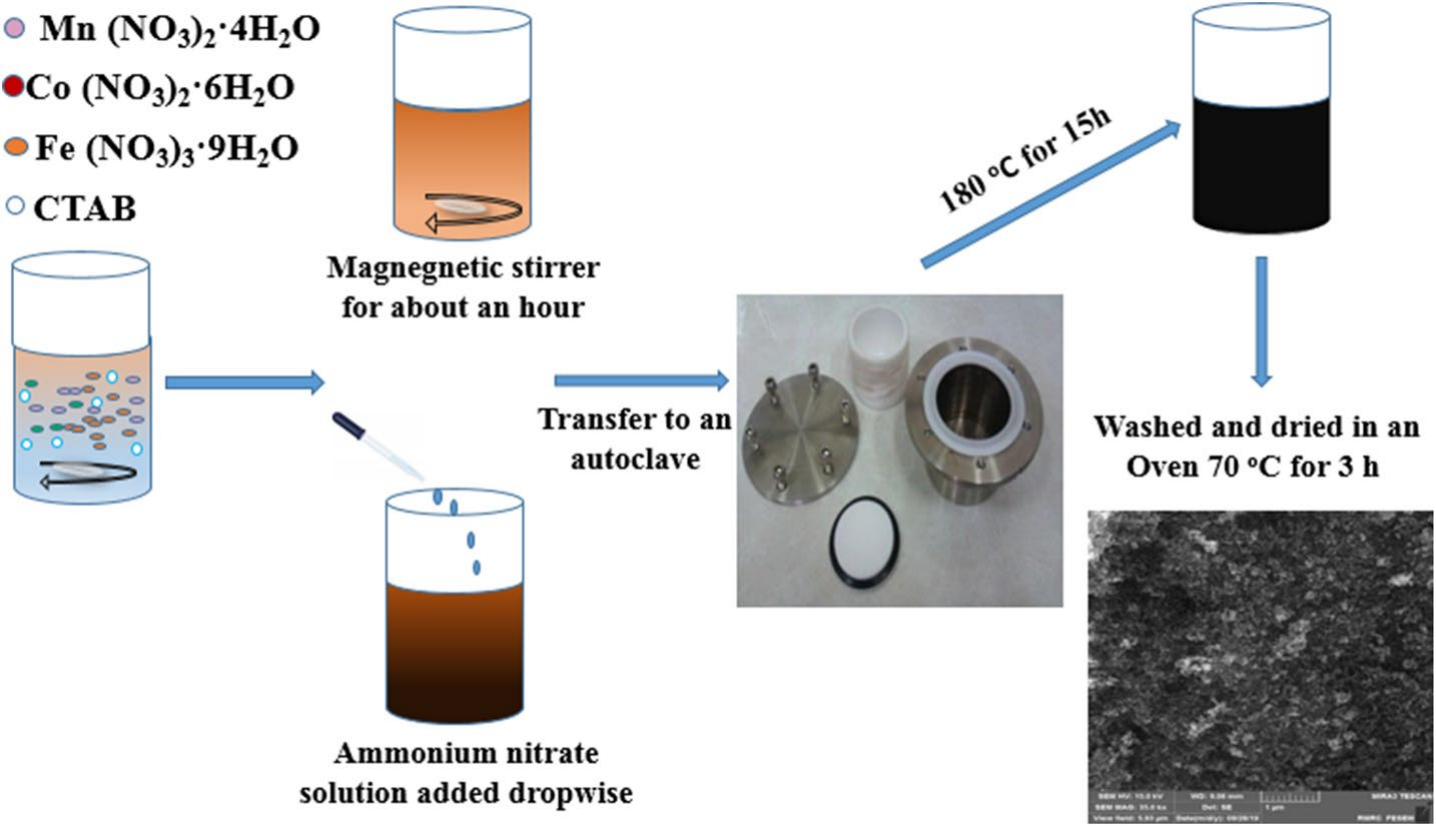
A schematic of the procedure followed to synthesize the manganese/nickel ferrite nanoparticles.

All chemicals, including Mn(NO_3_)_2_.4H_2_O, Fe(NO_3_)_3_.9H_2_O, Ni(NO_3_)_2_.6H_2_O, and cetyltrimethylammonium bromide (CTAB), were purchased from Merck Co. (> 98%) and used without any further purification. The nanoparticle powders of Ni_1−x_Mn_x_Fe_2_O_4_ (x = 0.0, 0.2, 0.4, 0.6, 0.8, and 1) were prepared using a hydrothermal method, similar to our recent work on supercapacitance properties of Ni_1−x_Co_x_Fe_2_O_4_ nanoparticles^26^. First, stoichiometric amounts of the materials, listed in Table 1, were dissolved in 40 ml deionized (DI) water by stirring for about 1 h. Next, 1 ml of 25% ammonia solution was added into the prepared nitrate mixture under vigorous stirring until its pH reaches ~ 9. Next, we transferred the obtained mixture into a Teflon-lined stainless autoclave that subsequently heated in an oven at 180 °C for 15 h. Then, we allowed the autoclave to cool in ambient air to room temperature. Finally, we washed the obtained product with DI water and ethanol for several times and dried it in an oven at 70 °C for 3 h. The prepared powders were used for further characterizations. A schematic of the various steps followed in our synthesis procedure is shown in Fig. 1.

**Table 1:** Amounts (g) of the precursors to synthesize Ni_x−1_Mn_x_Fe_2_O_4_ nanoparticles.

x	Mn(NO_3_)_2_·4H_2_O	Ni(NO_3_)_2_·6H_2_O	Fe(NO_3_)_3_·9H_2_O	CTAB
0.0	0.000	0.291	0.800	0.25
0.2	0.050	0.232	0.800	0.25
0.4	0.100	0.174	0.800	0.25
0.6	0.150	0.116	0.800	0.25
0.8	0.200	0.058	0.800	0.25
1.0	0.251	0.000	0.800	0.25

### Characterization techniques

X-ray diffractometry (XRD) was used to investigate the crystal structures of the prepared materials by employing a PANalytical X'pert MPD (Philips, Cu-K_α_ radiation source, λ = 1.54056 Å). Raman spectroscopy equipped with an Nd:YAG laser working at λ_ex_ = 532 nm at room temperature was employed to characterize the structure of the materials. Field-emission scanning electron microscopy (FESEM) was employed to observe the morphologies of the prepared materials by employing a MIRA3TESCAN-XMU microscope. The UV–Vis optical absorptions of the materials were recorded using a Unico, 4,802 spectrophotometer.

### Electrochemical measurements

A three-electrode electrochemical setup was used to study the electrochemical properties of the materials by utilizing a VSP-300 Multichannel Potentiostat/Galvanostat/EIS instrument (Bio-Logic Science Instruments). The electrolyte was 3 M KOH solution, the reference was an Ag/AgCl electrode, the counter electrode was a square-shaped Platinum sheet (1 cm^2^, 99.99%), and the substrate for the working electrodes was a square-shaped nickel foam (1 × 1 cm^2^) washed with ethanol, acetone, and DI water. The working electrode material was prepared from the active material, acetylene black, and polyvinylidene difluoride (PVDF), as a binder, with the weight ratio of 80:15:5 solved in N-Methyl-2-pyrrolidone (NMP). The prepared material was coated on a nickel foam substrate by a brush. Cyclic voltammetry (CV) tests were performed at different scan rates in the potential range of 0–0.4 V. The chronopotentiometry galvanostatic charge–discharge (GCD) tests were performed at different current densities. An asymmetric supercapacitor in a two-electrode setup was also assembled using the MnFe_2_O_4_ nanoparticles as the positive electrode, activated carbon (AC) as the negative electrode, and a filter paper wetted with the electrolyte as the separator to demonstrate the real-world application of the prepared materials. To prepare the AC electrode, a mixture of AC powder and PVDF, as a binder, was mixed with the weight ratio of 90:10 and solved in NMP. The prepared ink was coated on a nickel foam substrate by a brush and dried at 60 °C for 10 h.

### Computational methods

First-principles calculations were performed in the framework of density functional theory (DFT), as implemented in the Quantum Espresso package (version 6.2)^34^, using the plane-wave basis set and ultrasoft pseudopotentials^35^ and the valence electrons included Ni 3d 4 s, Mn 3d 4 s, Fe 3d 4 s, and O 2 s 2p states. Spin polarization was included in both geometry optimizations and electronic structure calculations. The generalized gradient approximation (GGA) developed by Perdew, Burke, and Ernzerhof (PBE)^36^ was applied for electron exchange–correlation functionals with the on-site Coulomb repulsion U terms^37^ of U(Ni) = 6.2 eV, U(Mn) = 3.9 eV, and U(Fe) = 5.3 eV to reproduce experimental data. The kinetic energy cutoffs for wavefunctions and charge densities were set to 50 and 450 Ry, respectively, and the k-point grid of 6 × 6 × 5 was adopted for sampling the first Brillouin zone (BZ) for electronic structure calculations. All structures were fully relaxed until the convergence criteria of energy and force became less than 10^–6^ Ry and 10^–3^ Ry/Bohr, respectively. All crystal images and simulated XRD patterns were produced by VESTA^38^.

## Results and discussions

**Figure 2:**
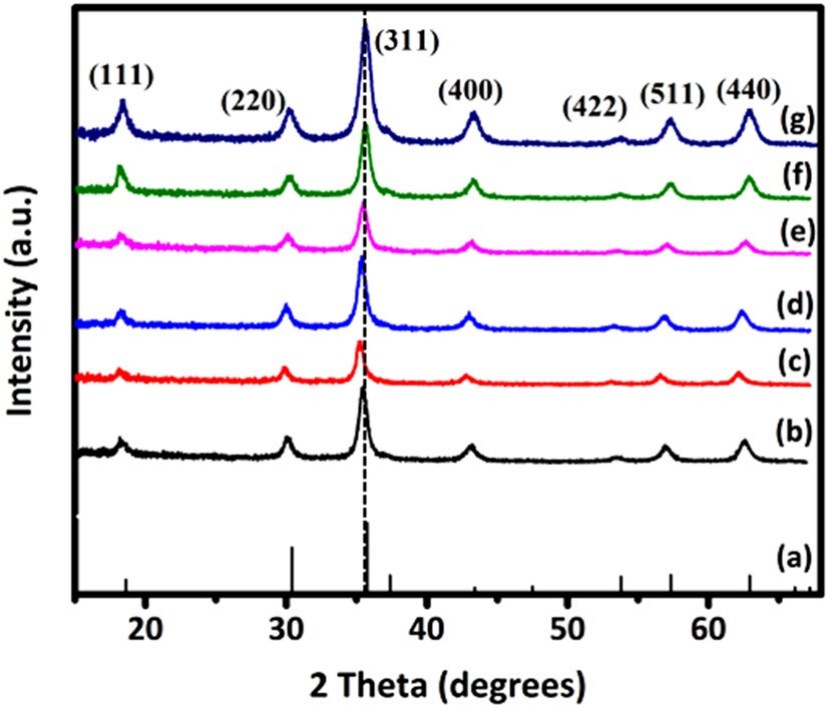
(**a**) Diffraction peaks corresponding to the JCPDS card #96-591-0,064 and XRD patterns of Ni_1−x_Mn_x_Fe_2_O_4_ nanoparticles where (**b**) x = 0, (**c**) x = 0.2, (**d**) x = 0.4, (**e**) x = 0.6, (**f**) x = 0.8, and (**g**) x = 1.

**Figure 3:**
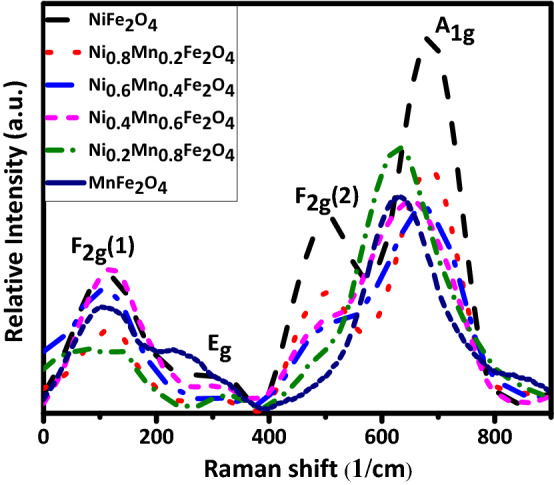
Raman spectra of the Ni_1−x_Mn_x_Fe_2_O_4_ nanoparticles.

XRD patterns of the prepared Ni_1−x_Mn_x_Fe_2_O_4_ samples, shown in Fig. 2, show all characteristic peaks of a ferrite material. The peaks are indexed as (111), (220), (311), (400), (422), (511), and (440) diffraction planes that are matched with the JCPDS card No. 96-591-0,064, indicating the cubic spinel structure with the space group of $\mathrm{F}\mathrm{d}\stackrel{-}{3}\mathrm{m}$. The patterns do not exhibit any additional peak, confirming the purity of the ferrite samples. The peaks are somewhat broad, which is due to the small size of the formed crystallites. The Williamson–Hall analysis was used to calculate the crystallite size and the lattice strain according to Eq. (1) for all intense peaks^39^:

1 $${\beta }_{hkl }\mathit{cos}{\theta }_{hkl}=\frac{k\lambda }{D}+4\varepsilon \mathit{sin}{\theta }_{hkl}$$

where θ_hkl_ and β_khl_ are the diffraction angle and the full width at half maximum (FWHM) of the (*hkl*) diffraction peak, *k* is the shape factor (here, 0.94), λ is the wavelength of the X-ray radiation source (1.54056 Å), and *D* and *ε* correspond to the crystallite size and the lattice strain, respectively. Accordingly, *D* and *ε* were obtained from the Y-intercept and the slope of the line from the plot of *4Sinθ* versus *βCosθ*^40^, respectively, as reported in Table 2. The lattice parameter *a*, also reported in Table 2, was calculated using Eq. (2) for the most intense peak^41^:

2 $${d}_{khl}=\frac{a}{\sqrt{{h}^{2}+{l}^{2}+{k}^{2}}}$$

where d_hkl_ is the inter-planar spacing for the most intense peak (311). It is seen that the lattice parameter *a* increases as the content of Mn^+2^ ions increases, which could be attributed to the larger ionic radius of Mn^+2^ (0.8 Å) than Ni^+2^ (0.69 Å). It is also seen that MnFe_2_O_4_ has both a larger crystallite size and a higher compressive strain than NiFe_2_O_4_, which is because of the stronger bonds that Mn^+2^ ions can form, as confirmed by Raman spectra (Fig. 3).

**Table 2:** Lattice parameter *a*, inter-planar spacing *d*, FWHM, crystal size *D*, strain *ε*, unit cell volume *V*, A_1g_ Raman shift for the Ni_1−x_Mn_x_Fe_2_O_4_ samples.

x	d (Å)	a (Å)	V (Å^3^)	FWHM (degrees)	D (nm)	ε	A_1g_ Raman shift (1/cm)
0.0	2.52	8.36	584.28	0.7233	9.36	− 0.00234	687.81
0.2	2.52	8.37	586.38	0.6436	9.44	− 0.00253	686.38
0.4	2.52	8.39	590.59	0.7460	9.12	− 0.00265	677.77
0.6	2.53	8.39	590.59	0.7350	8.39	− 0.00302	654.37
0.8	2.55	8.45	603.35	0.6441	10.13	− 0.00242	631.21
1.0	2.56	8.48	609.80	0.3936	9.54	− 0.00284	630.98

Raman spectra of the Ni_1−x_Mn_x_Fe_2_O_4_ nanoparticles are shown in Fig. 3. The A_1g_ band is due to the symmetric stretching of oxygen atoms along Fe–O (or M–O) tetrahedral bonds, the F_2g_(1) band is due to the translatory movement of the whole tetrahedron (FeO_4_), the F_2g_(2) band is due to the asymmetric stretching of Fe/M–O bonds, and the E_g_ band is due to the asymmetric and symmetric bending of O with respect to Fe^42^. The inverse spinel structure of the samples is confirmed by the Raman analysis^43^. As it is seen in Fig. 3 and Table 2, the A_1g_ peak shifts toward a lower frequency (from 687 to 630 1/cm) and the intensity of the F_2g_(2) peak decreases as Ni^+2^ ions are substituted with Mn^+2^ ions, consistent with differences between the Raman spectra of inverse and normal spinel structures^44^. This is due to the smaller ionic radius of Ni^+2^ than Mn^+2^. As a result, when Ni^+2^ ions are substituted with Mn^+2^ ions the lengths of bonds between the cations with the host atoms increase, leading to stronger interactions between atoms^45,46^. The experimentally obtained Raman modes are consistent with those for Ni_0.75_Zn_0.25_Fe_2_O_4_^42^, Mn_x_Fe_3−x_O_4_^47^, and ZnCuFe_2_O_4_^48^.

**Figure 4:**
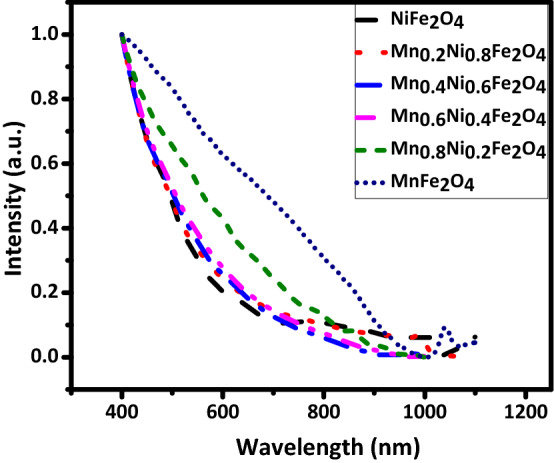
Absorption spectra of the Ni_1−*x*_Mn_*x*_Fe_2_O_4_ samples.

**Figure 5:**
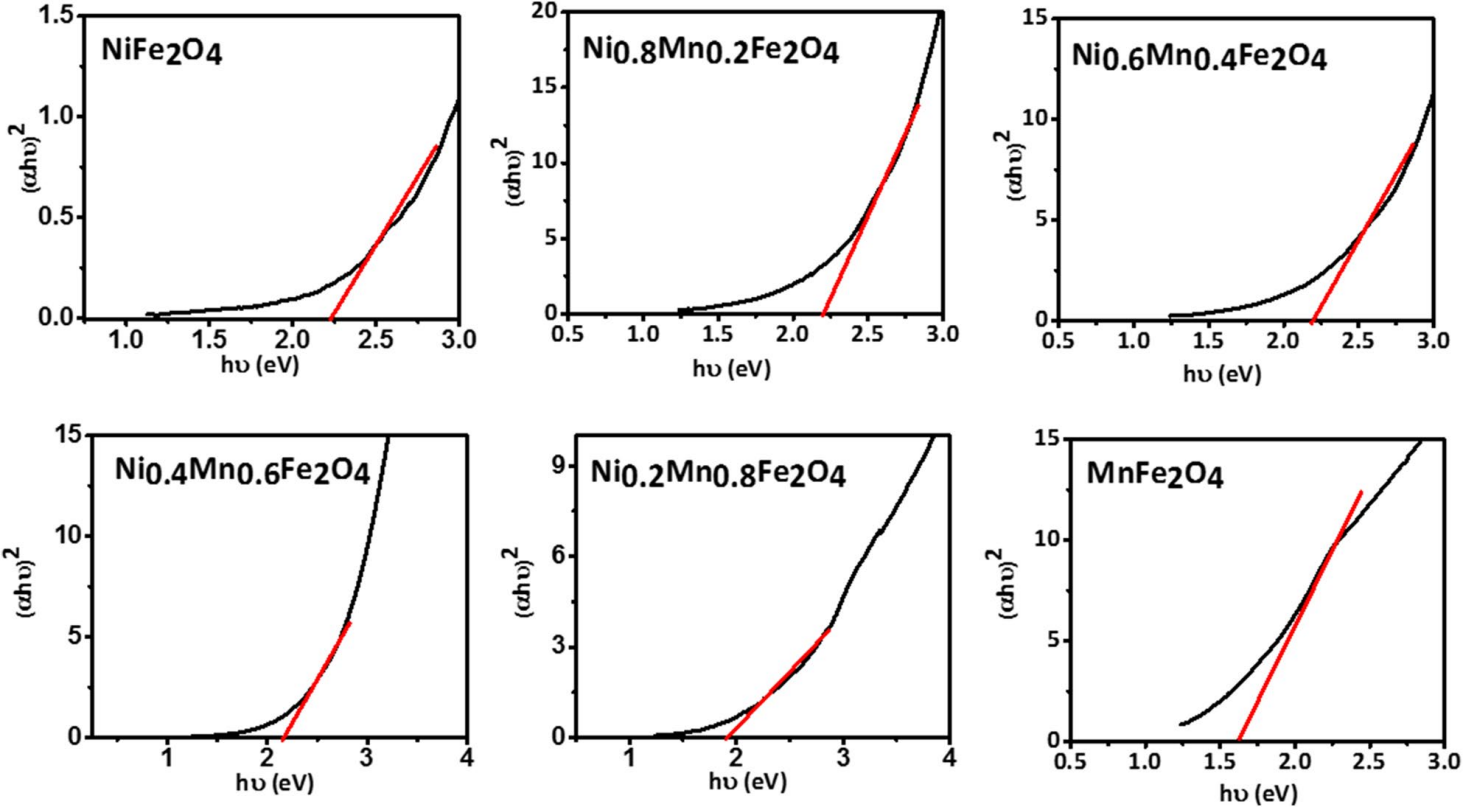
Tauc plots of the Ni_1−*x*_Mn_*x*_Fe_2_O_4_ nanoparticles assuming direct bandgaps.

**Figure 6:**
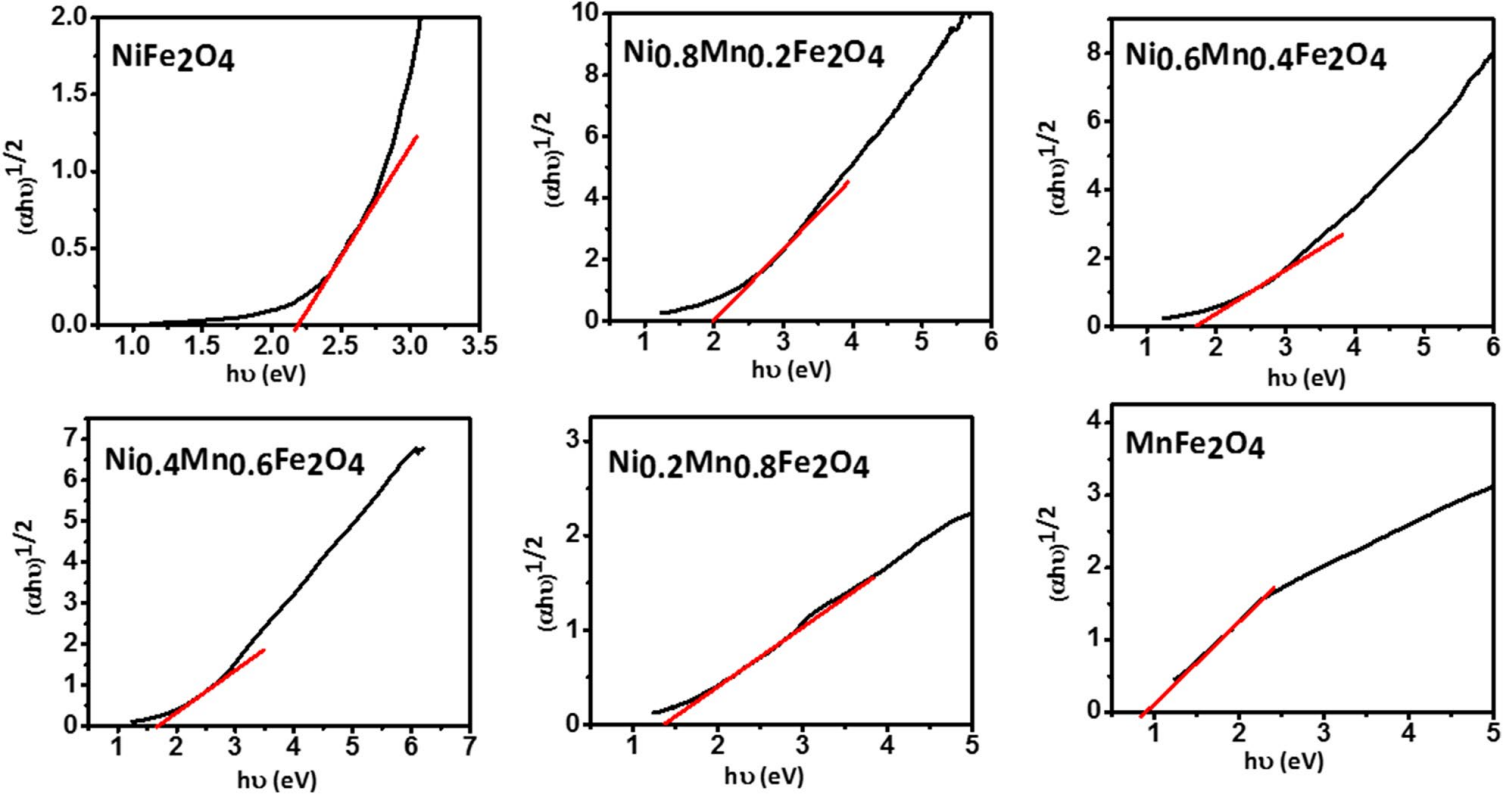
Tauc plots of the Ni_1−*x*_Mn_*x*_Fe_2_O_4_ nanoparticles assuming indirect bandgaps.

Figure 4 shows the optical absorption spectra of the prepared Ni_1−x_Mn_x_Fe_2_O_4_ nanoparticles. It is seen that as the content of Mn^+2^ increases the absorption edge undergoes a redshift and the absorbance is enhanced in both visible and near-infrared regions. These absorption spectra are consistent with those of previous reports on crystalline Mn_1–x_Ni_x_Fe_2_O_4_^49^ and Mn_1−x_Co_x_Fe_2_O_4_^50^. It is insightful to find how the incremental substitution of Ni with Mn in Ni_1−x_Mn_x_Fe_2_O_4_ will affect its bandgap. Because generally, the lower the bandgap the higher the specific capacitance. The optical bandgap *E*_*g*_ can be determined using the Tauc's relation in Eq. (3) ^51^

3 $$\alpha \mathrm{h}\nu = \mathrm{A}(\mathrm{h}\nu - \mathrm{E}\mathrm{g})\mathrm{n}$$

where *α* is the absorbance, h is the Plank's constant, ν is the light frequency, A is a proportionality constant, *E*_*g*_ is the optical bandgap, and n is a constant that depends on the bandgap type (1/2 and 2 for direct and indirect bandgaps, respectively). The optical bandgap can be estimated from the intersection of a line drawn on the linear part of the plot of (αhυ)^n^ versus the photon energy hυ, known as the Tauc's plot, with the hυ axis. Tauc plots of the Ni_1−x_Mn_x_Fe_2_O_4_ nanoparticles are shown in Figs. 5 and 6 assuming direct and indirect bandgaps, respectively^46,52,53^. It is seen that both direct and indirect bandgaps decrease as the content of Mn^+2^ increases. It is known that the bandgap value is affected by various factors such as the crystallite size, structural parameters, and impurities. In this case, due to the larger radius of Mn^+2^ ions than Ni^+2^ ions, the ionic interactions get stronger as the content of Mn^+2^ increases. This, along with the doping-induced states, induces inner bands in the bandgap, providing additional paths between the conduction band and the valence band that decrease the bandgap value. This could indicate the better specific capacitance of the Mn-doped nanoparticles.

**Figure 7:**
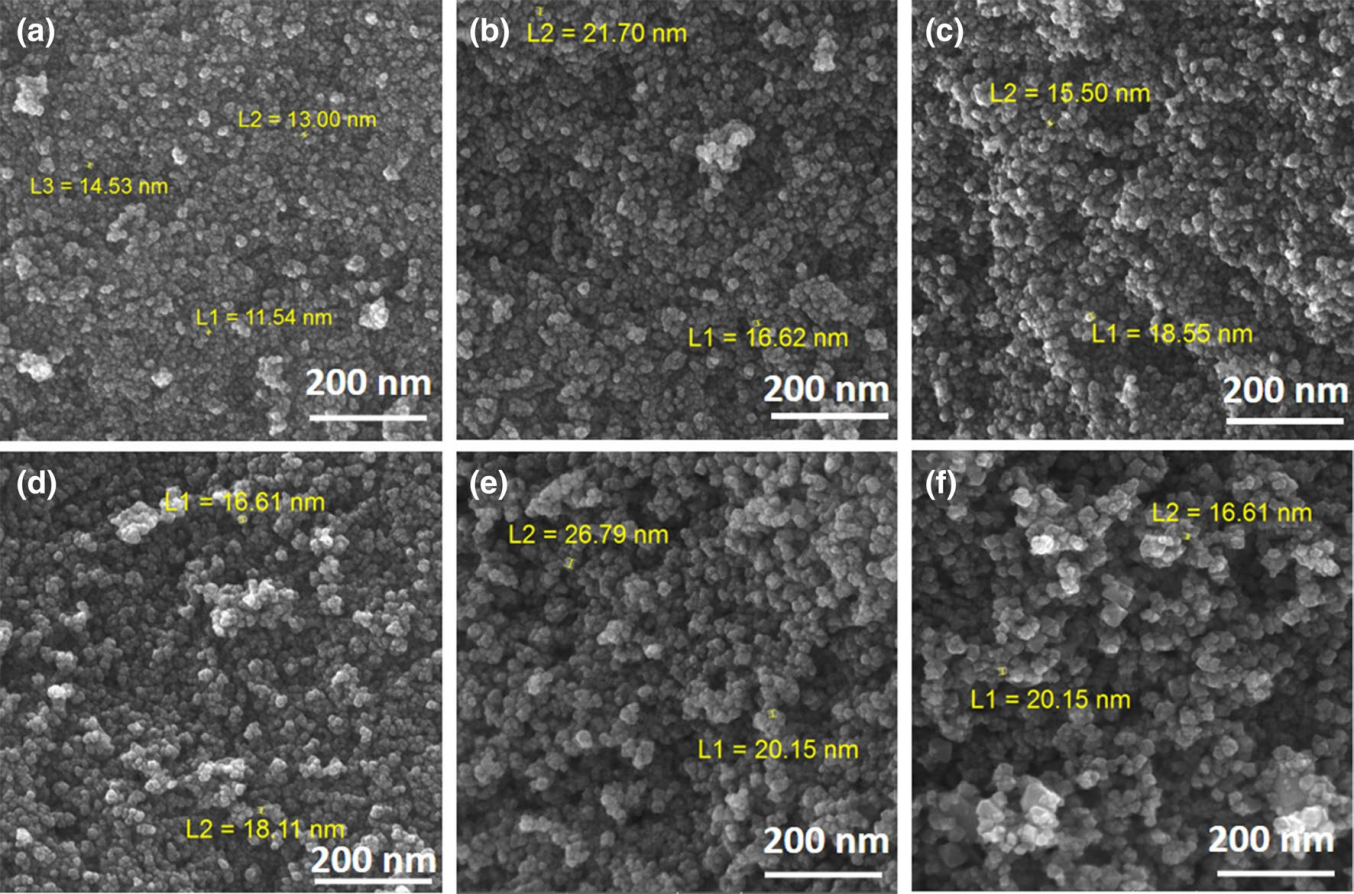
FESEM images of the prepared Ni_1−x_Mn_x_Fe_2_O_4_ nanoparticles, where (**a**) x = 0, (**b**) x = 0.2, (**c**) x = 0.4, (**d**) x = 0.6, (**e**) x = 0.8, and (**f**) x = 1.

FESEM images of the prepared Ni_1−x_Mn_x_Fe_2_O_4_ nanoparticles are shown in Fig. 7. The images exhibit highly agglomerated and spherical nanoparticles with a small grain size thanks to the use of CTAB^26^. It is seen that the average grain size increases when Ni^+2^ ions are substituted with Mn^+2^ ions, which is due to the larger ionic radius of Mn^+2^ ions than Ni^+2^ ions.

**Figure 8:**
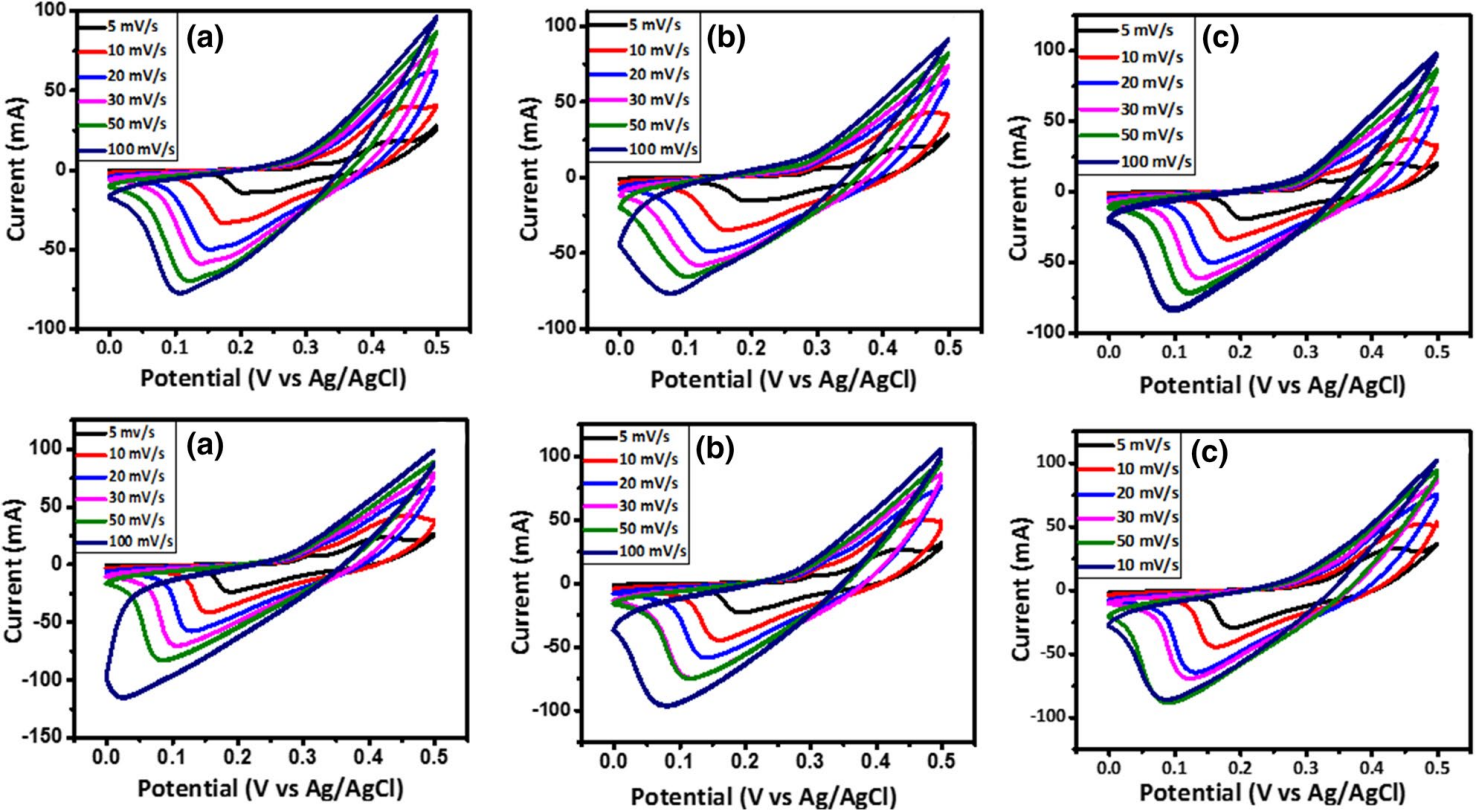
CV curves of the electrodes constructed from the prepared Ni_1−x_Mn_x_Fe_2_O_4_ nanoparticles where (**a**) x = 0, (**b**) x = 0.2, (**c**) x = 0.4, (**d**) x = 0.6, (**e**) x = 0.8, and (**f**) x = 1 at the scan rates of 5–100 mV/s.

**Figure 9:**
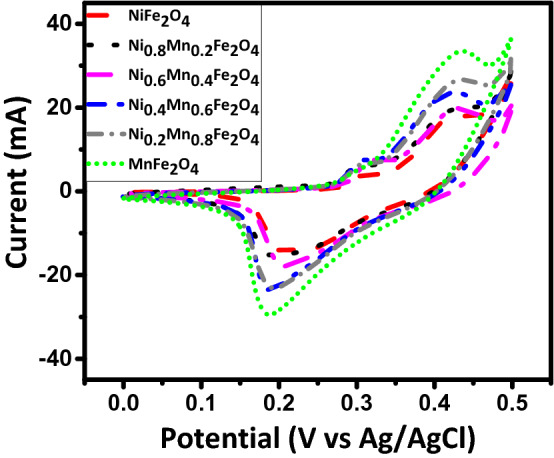
Comparative CV curves of the electrodes constructed from the prepared Ni_1−x_Mn_x_Fe_2_O_4_ nanoparticles at the scan rate of 5 mV/s.

Cyclic voltammetry (CV) tests were carried out within the potential range of 0.0–0.5 V for three-electrode tests with scan rates varying from 5 to 100 mV/s, as shown in Fig. 8. The CV curves display faradic currents, which are generated by the reduction or oxidation of some chemical active materials at the electrode. Accordingly, there are two peaks in the CV curves: (1) the oxidation peak in positive currents and (2) the reduction peak in negative currents. The oxidation and reduction peaks shift to higher and lower potentials as the scan rate increases. It is well known that the area within a CV curve is directly proportional to its specific capacitance. Thus, it is seen in all of the CV curves that the specific capacitance decreases as the scan rate increases^25^. Because at high scan rates, the electrolyte ions do not have enough time to diffuse entirely into the electrode nanopores wherever the faradaic reactions occur, making some part of the active surface areas inaccessible. Furthermore, this could be attributed to the existence of a large ohmic resistance at high scan rates^25,26,31^. The comparative CV curves of the samples at the scan rate of 5 mV/s are also shown in Fig. 9. Based on the area within the CV curves, it is evident that the incremental substitution of Ni with Mn leads to the enhancement of the specific capacitance of the Ni_1−x_Mn_x_Fe_2_O_4_-based electrodes, resulting from the decreased bandgap of Ni_1−x_Mn_x_Fe_2_O_4_ nanoparticles for a higher x, which itself enhances faradaic reactions at the electrode surface and enhances the specific capacitance.

**Figure 10:**
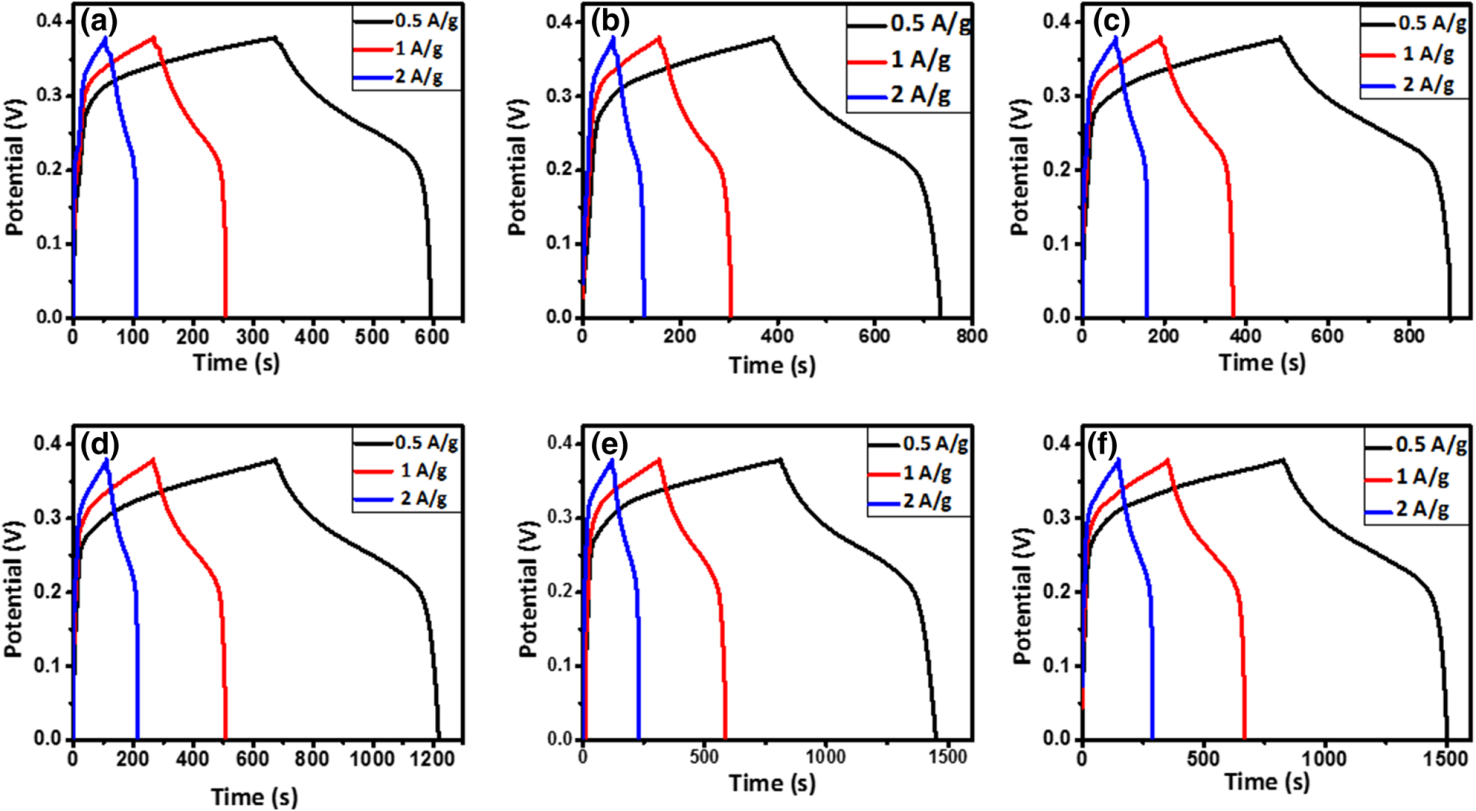
GCD curves of the electrodes constructed from the prepared Ni_1−x_Mn_x_Fe_2_O_4_ nanoparticles where (**a**) x = 0, (**b**) x = 0.2, (**c**) x = 0.4, (**d**) x = 0.6, (**e**) x = 0.8, and (**f**) x = 1 at the current densities of 5–100 mV/s.

**Figure 11:**
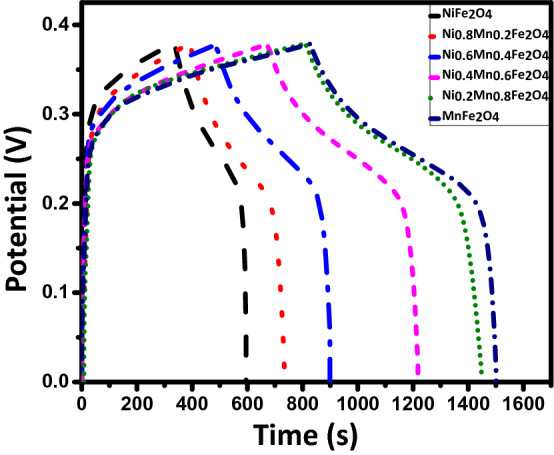
GCD curves of the electrodes constructed from the prepared Ni_1−x_Mn_x_Fe_2_O_4_ nanoparticles at the current density of 0.5 A/g.

**Figure 12:**
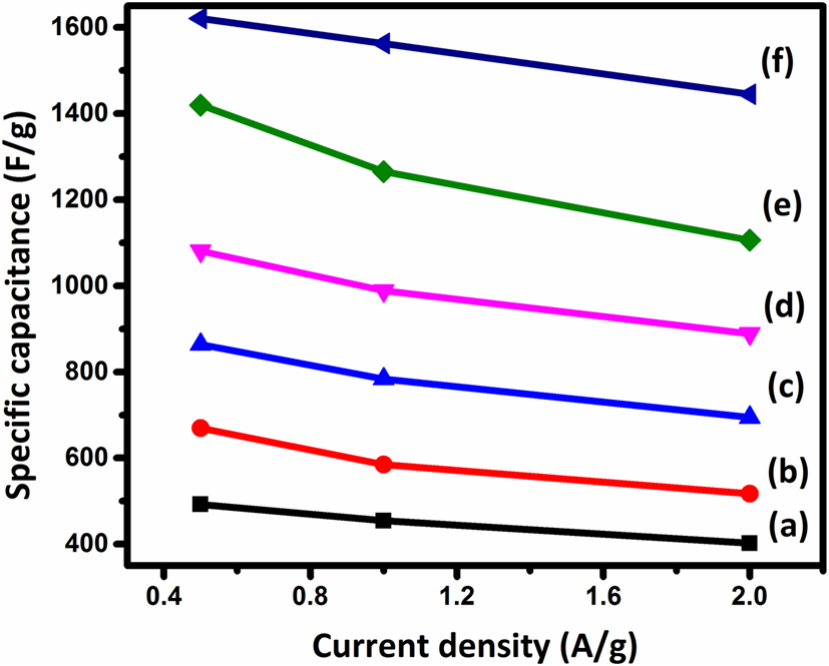
Specific capacitance versus current density for the electrodes constructed from the prepared Ni_1−x_Mn_x_Fe_2_O_4_ nanoparticles where (**a**) x = 0, (**b**) x = 0.2, (**c**) x = 0.4, (**d**) x = 0.6, (**e**) x = 0.8, and (**f**) x = 1.

**Figure 13:**
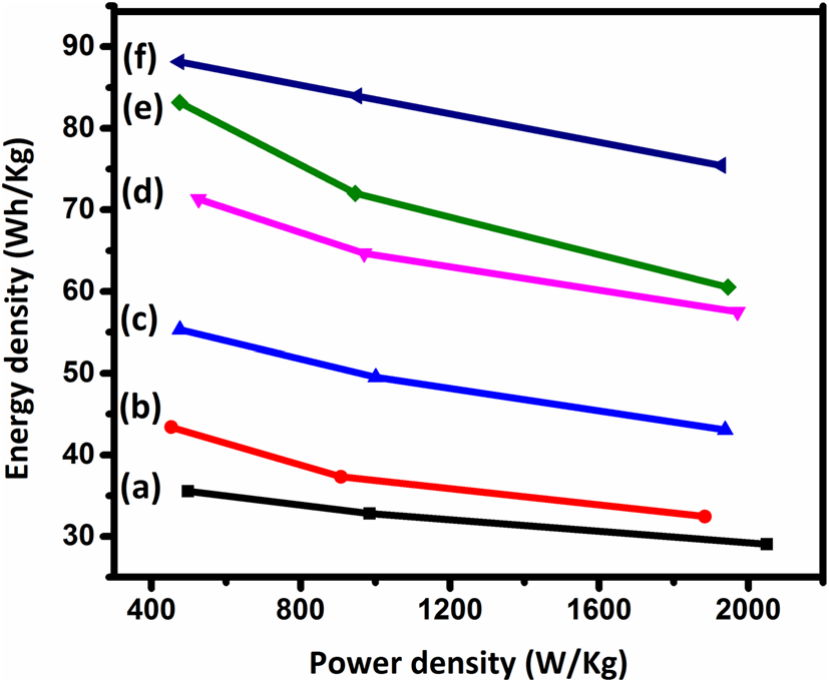
Ragone (energy density versus power density) plots for the electrodes constructed from the prepared Ni_1−x_Mn_x_Fe_2_O_4_ nanoparticles where (**a**) x = 0, (**b**) x = 0.2, (**c**) x = 0.4, (**d**) x = 0.6, (**e**) x = 0.8, and (**f**) x = 1.

Galvanostatic charge–discharge (GCD) curves of the samples at different current densities for three-electrode tests are shown in Fig. 10. The nonlinear discharge curves show that the capacitive performance results from both the electric double-layer capacitance and the pseudocapacitance^31^. The potential drop observed at the beginning of the discharge curve indicates both the very low internal series resistance (R_s_) of the prepared electrodes in the KOH electrolyte and the low contact resistance at the interface of the current collector and the electrolyte^25^. It is seen that the discharge time decreases as the current density increases, which is due to the lower accessibility of pores in the active material for electrolyte ions at higher currents^16^. The comparative GCD curves of the samples at the current density 0.5 A/g are also shown in Fig. 11. It is seen that the discharge time increases as Ni is incrementally substituted with Mn in Ni_1−x_Mn_x_Fe_2_O_4_-based electrodes. The specific capacitance values were calculated from Eq. (4):

4 $${C}_{sp}=2I\frac{\int Vdt}{m{(\Delta V)}^{2}}$$

where C_sp_ is the specific capacitance (F/g), I/m is the current intensity (A/g), $\int Vdt$ is the area under the discharge curve, and ΔV is the active potential window^54–56^. The electrode based on the MnFe_2_O_4_ nanoparticles shows the highest capacitance of 1,221 F/g at the current density of 0.5 A/g. Energy densities and power densities of the samples were also calculated using Eqs. (5) and (6)^55^:

5 $$\mathrm{E}\mathrm{n}\mathrm{e}\mathrm{r}\mathrm{g}\mathrm{y} \,\mathrm{d}\mathrm{e}\mathrm{n}\mathrm{s}\mathrm{i}\mathrm{t}\mathrm{y} =\frac{1}{2}{C}_{sp}{(\Delta \mathrm{V})}^{2}$$

6 $$\mathrm{P}\mathrm{o}\mathrm{w}\mathrm{e}\mathrm{r}\, \mathrm{d}\mathrm{e}\mathrm{n}\mathrm{s}\mathrm{i}\mathrm{t}\mathrm{y} = \frac{Energy\, density}{t}$$

where t is the discharge time. The specific capacitance values and the energy and power densities of the samples are reported in Table 3. As can be seen in Fig. 12, the specific capacitance decreases as the current density increases, which can be explained by considering the ion diffusion mechanism. In other words, at a lower current density, the electrolyte ions have enough time to access the highest number of active sites on the electrode material, leading to a higher specific capacitance^54^. According to Table 3, the specific capacitance of Ni_1−x_Mn_x_Fe_2_O_4_-based electrodes increases considerably as the content of Mn increases, consistent with CV curves in Fig. 9, as discussed above. The Ragone plots of the sample are shown in Fig. 13, indicating that the MnFe_2_O_4_-based electrode exhibits the remarkable specific energy density of 35.55 Wh/kg with the specific power density of 479.92 W/kg, which are much higher than those of the other samples.

**Table 3:** Specific capacitances, energy densities, and power densities of the electrodes constructed from the prepared Ni_1−x_Mn_x_Fe_2_O_4_ nanoparticles at the current density of 0.5 A/g.

x	Specific capacitance (F/g)	Energy density (Wh/kg)	Power density (W/kg)
0.0	492	35.55	497.92
0.2	601	43.41	452.98
0.4	766	55.31	476.35
0.6	988	71.34	526.25
0.8	1,152	83.18	475.28
1.0	1,221	88.16	473.96

**Figure 14:**
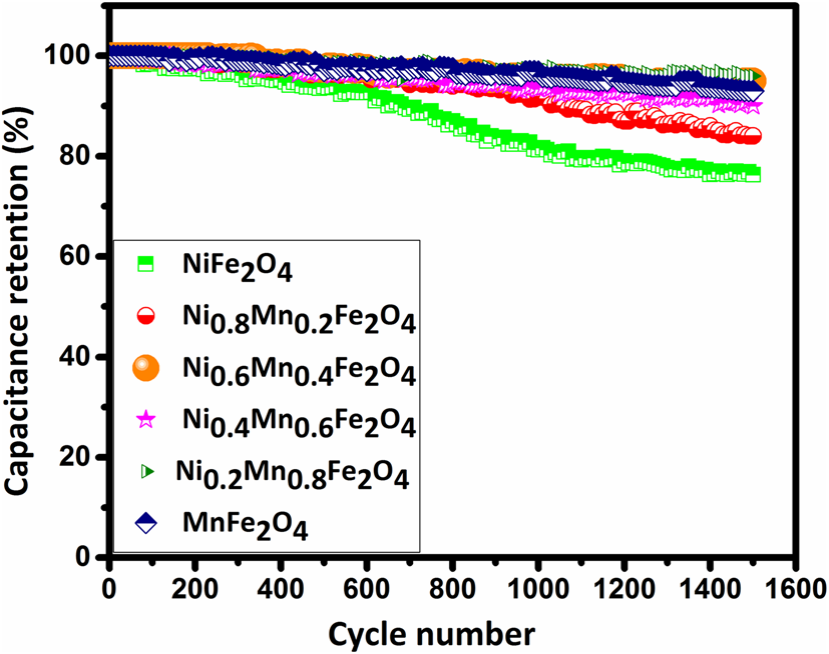
Cycling stabilities of the electrodes constructed from the prepared Ni_1−x_Mn_x_Fe_2_O_4_ nanoparticles over 1,500 GCD cycles at the current density of 3 A/g.

Cyclic stability tests were performed at the current density of 3 A/g for 1,500 GCD cycles, as shown in Fig. 14. It is seen that the substitution of Ni with Mn in Ni_1−x_Mn_x_Fe_2_O_4_-based electrodes considerably improves their cycling stability. This is another strength of MnFe_2_O_4_, in addition to its higher specific capacitance, as compared to NiFe_2_O_4_ for real-world commercial supercapacitor applications.

We observed that the electrode based on the prepared MnFe_2_O_4_ nanoparticles exhibited the highest specific capacitance and a very good stability. To demonstrate the real-world application of the electrode material, an asymmetric supercapacitor was assembled using the MnFe_2_O_4_ nanoparticles as the positive electrode, AC as the negative electrode (refer to the "Electrochemical measurements" section for the preparation method), and a filter paper wetted with the electrolyte as the separator. According to the specific capacitance of the AC electrode (150 F/g), and in order to achieve the maximum operating potential window and performance, the optimal mass ratio between the positive and negative electrodes ($\frac{{m}_{+}}{{m}_{-}}$) was balanced according to Eq. (7)^4,56,57^:

7 $$\frac{{m}_{+}}{{m}_{-}}=\frac{{C}_{S}^{-}{\Delta V}^{-}}{{C}_{s}^{+}{\Delta V}^{+}}$$

**Figure 15:**
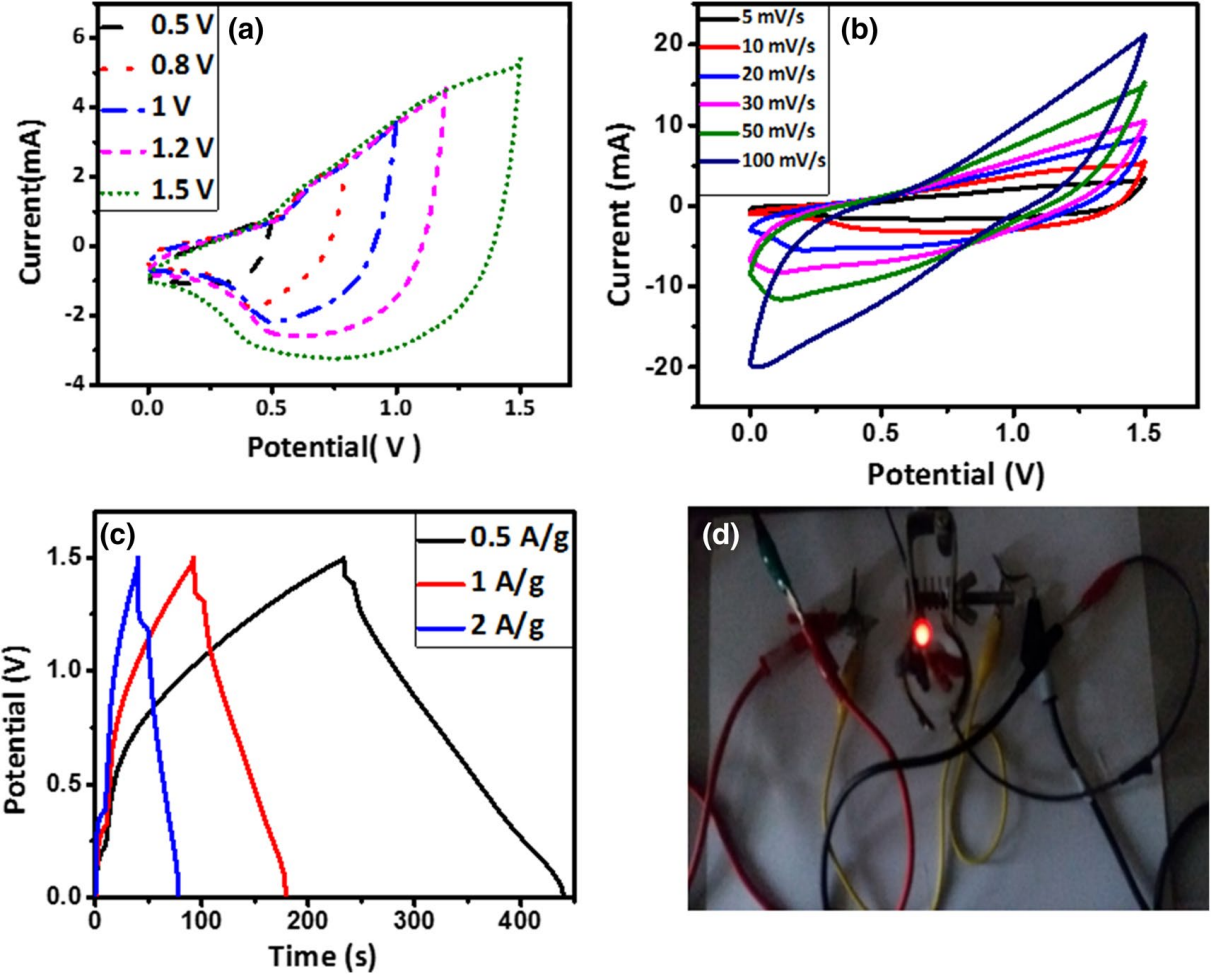
CV curves of the MnFe_2_O_4_//AC asymmetric device (**a**) at different potential windows at 10 mV/s and (**b**) at different scan rates. (**c**) GCD curves of the device at different current densities. (**d**) A picture of the assembled asymmetric supercapacitor lighting up a red LED.

Accordingly, the weight of AC powder was calculated ~ 4 mg. The CV curves of the electrode in the potential windows of 0.5–1.5 V and at different scan rates are shown in Fig. 15a and b, respectively. We chose the potential window of 1.5 V for further tests because of its highest CV area. It is seen that the CV curves retain their rectangular shape without apparent distortions up to 100 mV/s, indicating the high rate capability of this asymmetric supercapacitor. Interestingly, the asymmetric cell presents a wide and stable operating potential window up to 1.5 V in the KOH electrolyte that should afford high energy densities. The GCD curves of the electrode at different current densities are also shown in Fig. 15c. Figure 15d shows a picture of the assembled asymmetric supercapacitor lighting up a red LED, indicating the real-world application of the electrode material.

## DFT study

**Figure 16:**
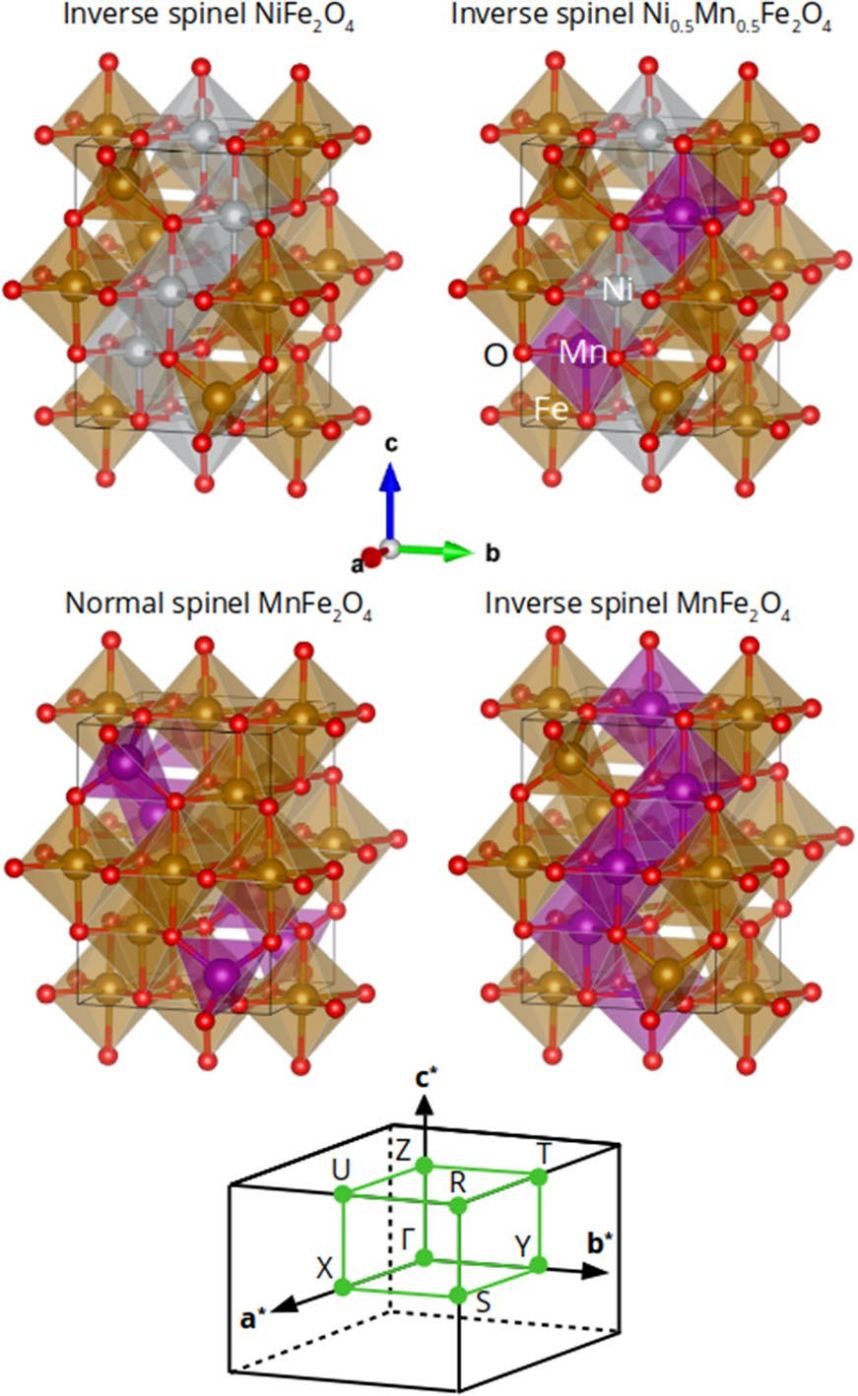
The considered unit cells of inverse NiFe_2_O_4_, inverse Ni_0.5_Mn_0.5_Fe_2_O_4_, and both normal and inverse MnFe_2_O_4_ spinel structures. The first Brillion zone and the k-path (in green) chosen to draw band structures are also shown in the bottom.

**Figure 17:**
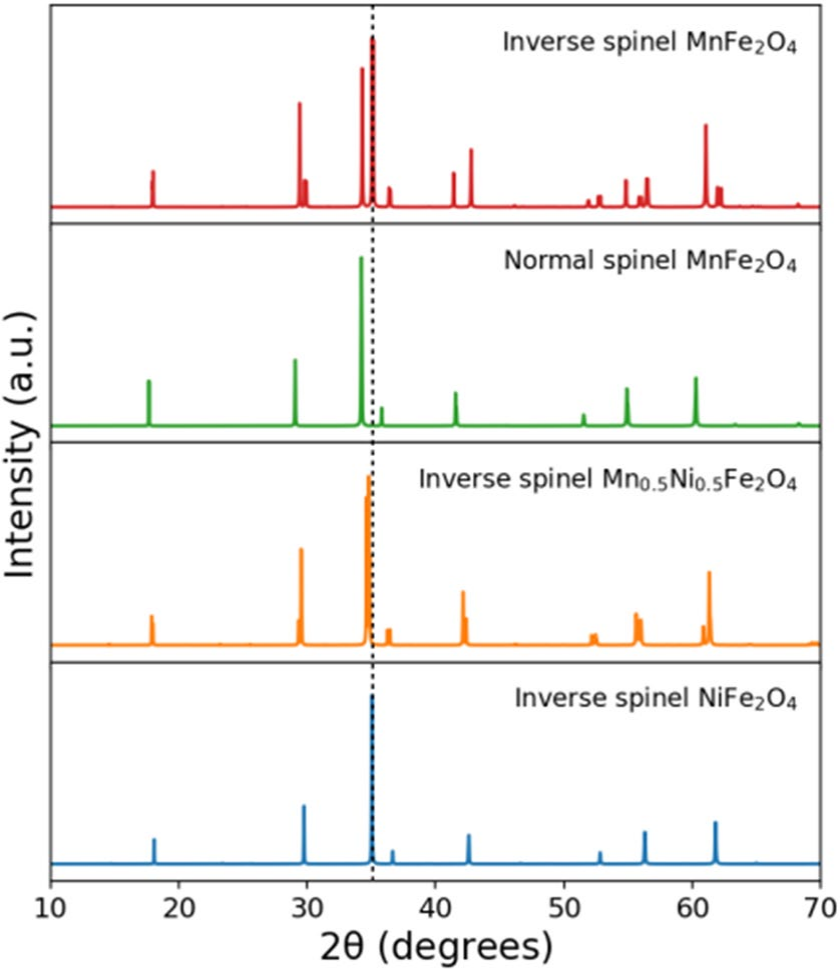
The computed XRD patterns of the crystal structures considered in this study.

**Figure 18:**
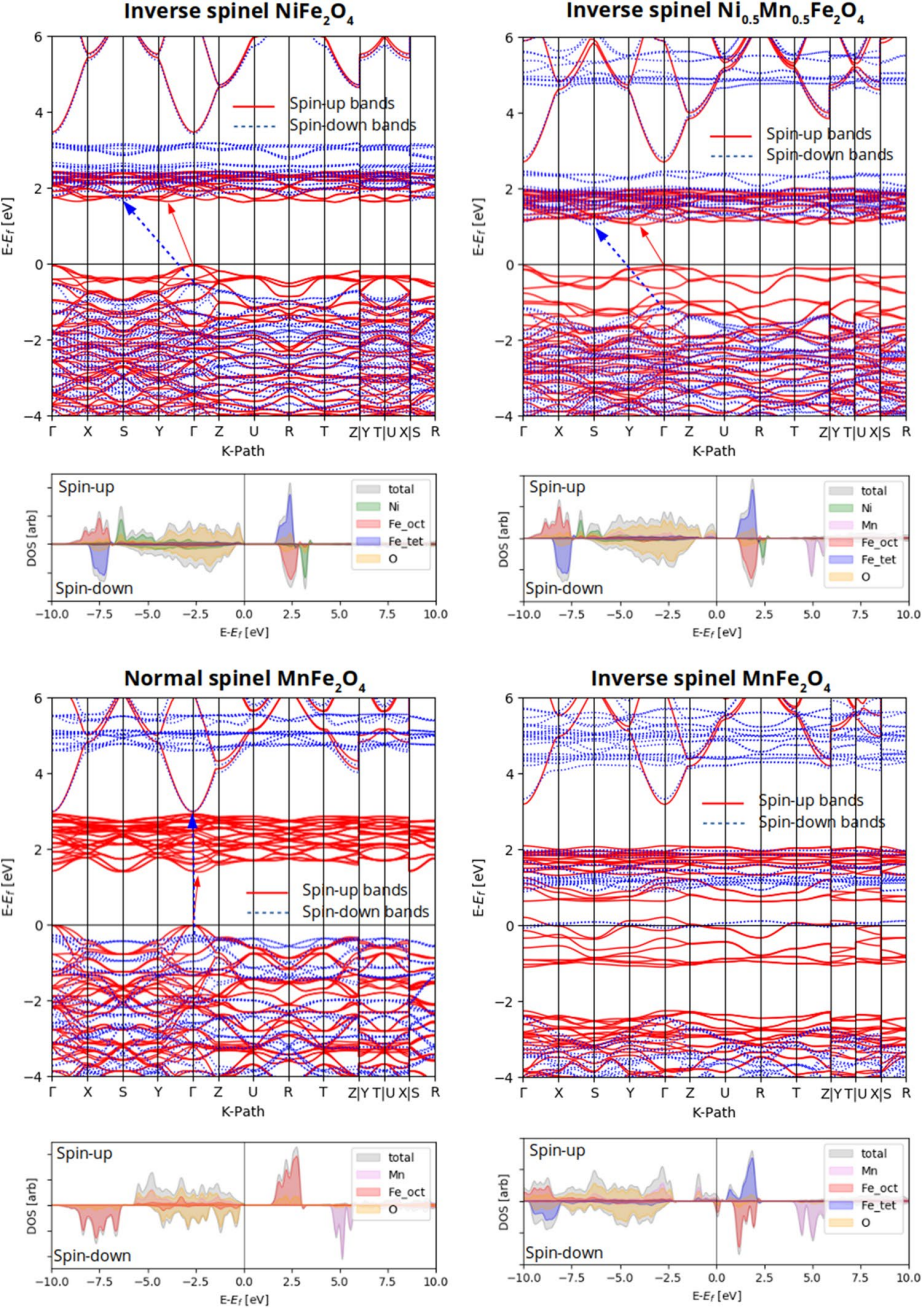
Electronic band structures and atom-projected density of states of the considered structures.

Nickel ferrite (NiFe_2_O_4_) and manganese ferrite (MnFe_2_O_4_) are both insulating, soft ferrimagnetic spinel ferrites. NiFe_2_O_4_ is configured in a fully inverse spinel structure, where Fe^3+^ ions occupy all the tetrahedral sites and half of the octahedral sites and Ni^2+^ ions occupy the other half of the octahedral sites^58^. On the other hand, MnFe_2_O_4_ crystallizes in a mixed-phase spinel structure^59^ with the almost low inversion degree of 0.2, where 80% and 20% of Mn^2+^ ions occupy the tetrahedral sites and octahedral sites, respectively, and Mn^2+^ and Fe^3+^ ions are distributed in the remaining tetrahedral and octahedral sites. Transport experiments have shown MnFe_2_O_4_ as an insulator with a small bandgap of 0.04–0.06 eV by^60^. To confirm the changes in geometrical and electronic properties of the samples, we performed a DFT study on NiFe_2_O_4_, Ni_0.5_Mn_0.5_Fe_2_O_4_, and MnFe_2_O_4_ structures. We considered 28-atom unit cells for all configurations as half of a simple cubic structure. Here, we considered an inverse spinel configuration for NiFe_2_O_4_ and Ni_0.5_Mn_0.5_Fe_2_O_4_. However, we considered both normal and inverse spinel configurations for MnFe_2_O_4_ (see Fig. 16). All the structures exhibited a ferrimagnetic character. To have a more clear understanding, we computed XRD patterns of the structure optimized by the DFT calculations, as shown in Fig. 17. It is seen that except inverse spinel MnFe_2_O_4_, the XRD patterns of all structures are largely similar to the experimentally obtained ones (Fig. 2). However, based on the shift in the position of the most-intense peak, it seems that the true XRD pattern of MnFe_2_O_4_ is a combination of normal and inverse spinel XRD patterns. Furthermore, it is seen that if one assumes the normal spinel structure for MnFe_2_O_4_, the changes in the lattice constants of a and c are consistent with the experimental XRD results (Fig. 2), confirming that the incremental substitution of Ni ions with Mn ions increases the cell volume. Figure 18 shows the electronic band structures and atom-projected density of states of the considered structures. It is seen that except inverse spinel MnFe_2_O_4_, the other structures are an insulator with different gaps for spin-up and spin-down states. However, when Co is incorporated into the structure, the structure becomes a conductor. The phenomenon and the increase of states near the Fermi level could help the structure to store charges, increasing the specific capacitance. It should be noted that the inverse spinel structure could not provide a true representation of the crystal structure of MnFe_2_O_4_, which predicts the structure as a conductor, which is not true experimentally. Lattice constants, spin-up gap, and spin-down gap of the considered structures calculated by DFT are also listed in Table 4. Furthermore, according to the atom-projected DOSs of normal and inverse MnFe_2_O_4_ spinel structures in Fig. 18, and considering that the true structure of MnFe_2_O_4_ is a combination of 20% inverse and 80% normal spinel structures, it could be said that the density of states near the fermi level of MnFe_2_O_4_ is higher than those of NiFe_2_O_4_ and Ni_0.5_Mn_0.5_Fe_2_O_4_, which could lead to a higher conductivity critical to supercapacitor applications.

**Table 4:** Lattice constants, spin-up gap, and spin-down gap calculated by DFT.

	a (Å)	c (Å)	Spin-up gap (eV)	Spin-down gap (eV)
Inverse spinel NiFe_2_O_4_	6.00	8.49	1.6 (indirect)	2.2 (indirect)
Inverse spinel Ni_0.5_Mn_0.5_Fe_2_O_4_	6.08	8.52	1 (indirect)	2.1 (indirect)
Normal spinel MnFe_2_O_4_	6.14	8.68	1.4 (indirect)	4.5 (direct)
Inverse spinel MnFe_2_O_4_	5.96	8.71	0	0

## Conclusion

In summary, spinel Ni_1−x_Mn_x_Fe_2_O_4_ nanoparticles with x = 0, 0.2, 0.4, 0.6, 0.8, and 1 were synthesized by a simple hydrothermal method to study how the incremental substitution of Ni with Mn can affect their electrochemical properties as supercapacitor electrode materials. We observed that by substituting Ni with Mn, the inverse spinel structure of NiFe_2_O_4_ changed to the almost normal spinel structure of MnFe_2_O_4_ and the optical bandgap decreased, leading to both considerably enhanced electrochemical properties and cycling stability. The electrode based on the MnFe_2_O_4_ nanoparticles exhibited the highest specific capacitance of 1,221 F/g at the current density of 0.5 A/g, with the corresponding energy density and power density of 88.16 Wh/kg and 473.96 W/kg, respectively. The density functional theory study on the same structures confirmed the changes in their geometrical and electronic properties.

## Data Availability

The datasets generated during and/or analyzed during the current study are available from the corresponding author on reasonable requests.
